# Reaching people with undiagnosed HIV infection through assisted partner notification

**DOI:** 10.1097/QAD.0000000000001631

**Published:** 2017-11-01

**Authors:** Shona Dalal, Cheryl Johnson, Virginia Fonner, Caitlin E. Kennedy, Nandi Siegfried, Carmen Figueroa, Rachel Baggaley

**Affiliations:** a Department of HIV/AIDS, World Health Organization, Geneva, Switzerland; b Department of Psychiatry and Behavioral Sciences, Medical University of South Carolina, Charleston, South Carolina; c Department of International Health, Johns Hopkins University Bloomberg School of Public Health, Baltimore, Maryland, USA; d Independent Clinical Epidemiologist, Cape Town, South Africa.

The letter from Davey and Wall [1] on our article titled ‘Improving HIV test uptake and case finding with partner notification’ makes the case for the inclusion of couples HIV testing and counselling in partner notification approaches, and presents their preliminary results from this approach in South Africa.

One of the assisted partner notification approaches described in our review [2] includes dual referral, which we defined as a voluntary process ‘where the provider accompanies the index patient when they disclose their status and offers HIV testing services to their partner (s)’. Therefore, dual referral provides a conducive environment for couples counselling and testing to occur. Two of the studies included in our review offered dual referral as one partner notification option [3,4]. The authors correctly note that the randomized controlled trial by Rosenberg *et al.*[5], which we included in our systematic review, provided couples counselling following partner notification, and included a single invitation for a partner of each enrolled pregnant woman. The stated objective of our review was to assess the effectiveness of partner notification services; to do this, we defined and compared passive and assisted approaches. We did not evaluate the type (e.g. couples, provider-initiated, client-initiated and self-testing) or location (e.g. facility, community or home-based) of testing and counselling that was provided to partners of index patients.

We agree with the authors that couples counselling is a useful approach that should be offered within HIV testing services when trained counsellors are able to provide these services. WHO consolidated guidelines on HIV testing services [6], and guidance on couples HIV testing and counselling [7], support this with a strong recommendation for couples counselling. Some of the many benefits of partner and couples HIV testing include mutual support for sexual or injecting partners to access HIV prevention, treatment and care services; improved adherence and retention on treatment; increased support for the prevention of mother-to-child transmission; and the prioritization of effective HIV prevention for serodiscordant couples (condom use, antiretroviral therapy and preexposure prophylaxis for HIV-negative partners). However, despite the existing WHO recommendation to offer partner and couples testing, and its inclusion in the HIV polices of many countries, it has not often been actively prioritized, nor widely implemented. The WHO recommendation on assisted HIV partner notification [8] that was a result of our systematic review is in line with, and builds upon, the existing WHO recommendations.

Partner notification is a related, albeit different approach that does not require people to disclose their status to a partner/s with a counsellor as a couple, but rather includes it as one option among others. It is more encompassing of different types of partnerships, whether committed couples, or casual, or short-term partners. The underlying public health premise of partner notification is to offer HIV testing to people who may have been exposed to HIV infection either through sexual or injecting drug contacts, to reach those with undiagnosed HIV, and to prevent further transmission. The impetus therefore is to notify as many partners of their potential exposure as possible. Results from our review found that on average, the ratio of partners identified per index case through partner notification services was two (range 0.58–5.58), demonstrating the importance of encouraging index patients to identify all partners who may have been exposed. Furthermore, as no single approach to disclosure is acceptable to everyone, choice is important. People diagnosed with HIV should be given options for partner notification and be allowed to choose different methods for different partners, or to decline altogether. For example, they may want to use a passive approach to contact some partners, whom they feel comfortable notifying on their own, but may prefer the provider to assist them in contacting others.

In summary, WHO recommends a range of approaches to increase partner testing as an important way to reach people with undiagnosed HIV and link them to treatment. Couples testing, as recommended by WHO since 2012, is one of these approaches, and mutual disclosure can have additional benefits. WHO has now broadened this recommendation to support a range of partner notification and testing approaches and strongly encourages countries to routinely recommend these voluntary partner testing options to all people with HIV.

## Acknowledgements

### Conflicts of interest

There are no conflicts of interest.
